# Real-Time Social Support Through a Mobile Virtual Community to Improve Healthy Behavior in Overweight and Sedentary Adults: A Focus Group Analysis

**DOI:** 10.2196/jmir.1770

**Published:** 2011-07-14

**Authors:** Yoshimi Fukuoka, Emiko Kamitani, Kemberlee Bonnet, Teri Lindgren

**Affiliations:** ^1^Institute for Health and AgingDepartment of Social and Behavioral SciencesUniversity of California, San FranciscoSan Francisco, CAUnited States; ^2^Department of Community Health SystemSchool of NursingUniversity of California, San FranciscoSan Francisco, CAUnited States; ^3^Institute for Health and AgingUniversity of California, San FranciscoSan Francisco, CAUnited States; ^4^College of Nursing, Rutgers,the State University of New JerseyNewark, NJUnited States

**Keywords:** Mobile phone, physical activity, weight loss, diet, overweight, sedentary lifestyle, focus group, social support, diabetes mellitus

## Abstract

**Background:**

The onset of type 2 diabetes mellitus can be prevented or delayed by lifestyle changes. Communication technologies such as a mobile phone can be used as a means of delivering these lifestyle changes.

**Objectives:**

The purposes of this analysis were to explore applicability of potential components of a mobile phone-based healthy lifestyle program and to understand motivators and barriers to continued engagement in a mobile phone healthy lifestyle program.

**Methods:**

We conducted 6 focus groups (4 female and 2 male groups) in May and June 2010 with 35 focus group participants. The qualitative data were analyzed by 3 researchers using a qualitative description method in an ATLAS.ti software program. Inclusion criteria for enrollment in a focus group were as follows: (1) being aged from 30 to 69 years, (2) speaking and reading English, (3) having a sedentary lifestyle at work or during leisure time (screened by the Brief Physical Activity Survey questionnaire), and (4) having a body mass index (BMI) >25 kg/m^2^ (Asian >23 kg/m^2^) based on self-reported weight and height or 5) having a self-reported prediabetic condition.

**Results:**

The mean age was 51 (SD 10.6) years; 54% (n = 19) were white; 71% (n = 25) used a mobile phone at least once a week during the last month prior to the study enrollment; and mean BMI was 32.5 (SD 6.5) kg/m^2^. In the qualitative analyses, the following 4 major themes and their subthemes emerged: (1) real-time social support (real-time peer support from participants who are similarly engaged in a diet or physical activity program, and professional support from health care providers or a researcher), (2) tailoring of mobile phone programs (3) self-monitoring and motivation, and (4) potential barriers and sustainability of the program (fear of failing, age and mobile technologies, and loss of interest over time).

**Conclusions:**

Participants from a wide range of age and racial groups expressed interest in a mobile phone-based lifestyle program. Such a program that incorporates the themes that we identified may be able to help motivate participants to increase their physical activity and to improve their diet.

## Introduction

The rapidly growing incidence of diabetes is a serious worldwide public health concern. Approximately 7.8% of the population has diabetes and 18% is prediabetic in the United States [1]. The number of people with diabetes is expected to increase in the future. Intensive face-to-face lifestyle modification programs such as changing diets, increasing the level of physical activity, and losing a moderate amount of weight can prevent or delay the onset of type 2 diabetes [2,3]; however, these programs have been expensive to implement and sustain over time. Communication technologies are available to deliver and disseminate such lifestyle modification programs to much broader audiences, making them more cost effective, but we need to understand how to better apply these technologies [4].

The mobile phone has become one of the most personalized technologies in everyday life and is omnipresent across the world today. In the United States, 87% of adults own a mobile phone, and adoption rates are even higher in other global locations [5]. Mobile phone services and accessories include short message service (SMS), multimedia messaging service, internet access, Bluetooth technologies, and global positioning systems. These technologies and services can be incorporated into lifestyle modification programs. In addition, given the portability, affordability, availability, and feasibility of mobile phones, they can be used as a means to promote a healthy lifestyle (physical activity, diet, weight loss, etc) and to prevent or manage chronic illnesses.

Qualitative methodologies, including focus groups, have been used to evaluate feasibility and acceptability of existing mobile phone-based disease management or healthy lifestyle programs [6-8]. However, few studies have explored the individual needs and expectations of a mobile phone-based program to motivate and promote healthy lifestyle modifications in sedentary and overweight or obese adults. Understanding the user’s need and expectations is the first step toward designing an effective mobile phone-based healthy lifestyle program for individuals who are at risk of developing diabetes, as research has shown that a user-centered design is the best way to develop an effective lifestyle program [9]. Thus, we conducted 6 focus groups to explore the knowledge, attitudes, and behaviors of sedentary adults toward diabetes, diet, physical activity, weight loss, and the use of mobile phone-based healthy lifestyle programs in a diverse sample of sedentary overweight or obese women and men. The purposes of this analysis were to explore applicability of potential components of a mobile phone-based healthy lifestyle program and to understand motivators and barriers to continued engagement in a mobile phone healthy lifestyle program.

## Methods

### Study Design and Sample

Six focus groups (4 female and 2 male groups) were conducted in May and June 2010. Potential participants were recruited from urban communities through newspapers and flyers in hospitals. We screened 63 potential participants over the telephone, of whom 44 met all inclusion criteria. Of those who met all inclusion criteria, 35 participated in the focus group and 9 either did not show up for their scheduled focus group or had scheduling conflicts with potential focus group dates. The study was approved by the University of California San Francisco Institutional Review Board. All participants signed a written informed consent prior to the focus group study.

### Inclusion and Exclusion Criteria

The study inclusion criteria were (1) being aged from 30 to 69 years, (2) speaking and reading English, (3) having a sedentary lifestyle at work or during leisure time (screened by the Brief Physical Activity Survey questionnaire) [10], and (4) having a body mass index (BMI) >25 kg/m^2^ (Asian >23 kg/m^2^) based on self-reported weight and height or (5) having a self-reported prediabetic condition. Exclusion criteria were (1) known medical conditions or other physical problems requiring special attention in an exercise program, (2) severe hearing or speech problems, (3) current participation in a lifestyle modification program or research study, and (4) known eating disorder. We did not exclude people who had never used mobile phones or were not current mobile phone users, since a previous study indicated that both nonusers and users of mobile phones were able to effectively use mobile phone applications [10].

### Procedure

Individuals who were interested in participating in the focus group were screened over the telephone for inclusion and exclusion criteria. After the telephone screening, the potential participants were scheduled for a focus group. Focus groups were created based on gender and not on whether they owned or used mobile phones. Upon arrival for the focus group, participants gave written informed consent. Participants were assigned a focus group name that was associated with an alphanumeric identifier to ensure participant confidentiality; they then provided sociodemographic and medical information. The research nurse then measured participants’ weight, height, waist and hip circumferences, resting blood pressure, and hemoglobin A1c in a private office. Each focus group session began with brief introductions to promote comfort and sharing of ideas within the group. To initiate the discussion, prescripted, open-ended questions were used to explore knowledge, beliefs, and attitudes about diabetes, physical activity, diet, and weight control focusing on barriers and motivators in patients who are at a risk of developing diabetes. We then proceeded from a broad discussion of diabetes, physical activity, and diet to a more narrowly focused discussion of how a mobile phone program might be used by participants (Multimedia Appendix 1). Open-ended, “what-if” questions were used to elicit participants’ views on developing a useful and effective mobile phone-driven lifestyle change program. For example, we asked: “If you were going to use a mobile phone to motivate people to be physically active or eat a healthy diet, how would you use the phone?” Within these discussions, participants were asked for examples of possible applications, which led to more structured, investigator-driven prompts intended to further explore specific preconceived ideas of potential ways to use mobile phones to promote lifestyle behavior changes. For example, when participants stated that messages could be used to motivate them to continue the program, we asked: “If you were going to receive motivational messages via the mobile phone, what kind of messages would you like to receive?” This paper focuses on the participants’ ideas of how to use a mobile phone and their views of investigator-designed examples of a mobile phone-driven lifestyle change program. Participants were paid $20 at the end of the focus group. The focus group interviews were digitally recorded and transcribed verbatim by a professional transcriptionist. To ensure the fidelity of the transcription, we conducted spot checks of interviews, comparing the transcript with the digital recording.

### Data Management and Analysis

Descriptive statistics were used to describe the sample characteristics and medical history. These data were analyzed using SPSS 18.0 (IBM Corporation, Somers, NY, USA). Transcripts were imported into ATLAS.ti for Windows (version 6.0; ATLAS.ti GmbH, Berlin, Germany), a data management program designed to facilitate retrieval and organization of qualitative data for coding and analysis. Interview data were analyzed employing a qualitative description approach. Qualitative description was used to reduce and thematically analyze the semistructured interview data. Qualitative description involves a type of analysis that is “low inference” and less interpretive, not requiring a “highly conceptual or abstract rendering of data,” making it useful for researchers who wish to obtain “unadorned” or “minimally theorized” answers to specific questions [4]. Two investigators reviewed the transcripts independently and identified multiple codes based on significant dialogues raised from interview questions. These elements were clustered into categories by observed similarity. Data were then coded using hermeneutic methods to assign meaning to each category. As the final stage of analysis, larger meaningful themes were extracted from these categories, and then the relationship between themes was articulated.

## Results

### Sample Characteristics

The demographic characteristics of the participants are summarized in Table 1. The mean age was 51 (SD 10.6) years, ranging from 30 to 69 years; 46% (n = 16) of participants were minorities; 43% (n = 15) were never married; and 26% (n = 9) had a part-time or full-time job. During the last month prior to study enrollment, 71% (n = 25) of participants used a mobile phone at least once a week. The mean BMI was 32.5 (SD 6.5) kg/m^2^. The mean waist and hip circumferences were 106 (SD 14) cm and 117 (SD 16) cm, respectively.

**Table 1:** Sample demographics (N = 35)

Demographic characteristic		Mean (SD) or %(n)
Mean (SD)/(range) age (years)		51.0 (10.6)/(30–69)
Female gender		57% (20)
**Race/ethnic group**		
	White	54% (19)
	African American	31% (11)
	Asian	9% (3)
	Other/more than one race	6% (2)
Education: completed college or graduate school		40% (14)
**Marital status**		
	Never married	43% (15)
	Divorced/widowed	31% (11)
	Married/cohabiting	26% (9)
**Annual household income**		
	<$20,000	49% (17)
	$20,000–75,000	29% (10)
	>$75,000	11% (4)
	Don’t know/declined to answer	12% (4)
Part- or full-time employment		26% (9)
Used a mobile phone at least once a week during the last month prior to study enrollment		71% (25)
Current smoker		34% (12)
BMI kg/m^2^		32.5 (SD 6.5)
Resting blood pressure (mmHg)		126 (SD 14)
**Total cholesterol >200 mg/dL (self-report)**		
	Yes	43% (15)
	No	46% (16)
	Don’t know	12% (4)
**High density lipoprotein<40 mg/dL (self-report)**		
	Yes	29% (10)
	No	37% (13)
	Don’t know	34% (12)
Waist circumference (cm)		106 (SD 14)
Hip circumference (cm)		117 (SD 16)
Mean (SD) hemoglobin A1c (%)/range		5.7 (0.06) (5.1–7.4)

### Qualitative Data

Four major themes emerged from the focus group qualitative data analyses: (1) real-time peer social and professional support, (2) tailoring of timing, frequency, and content of messages in mobile phone programs, (3) combination of motivations, self-monitoring, and goal setting, and (4) potential barriers and sustainability of mobile phone programs. Each major theme consisted of specific subthemes. Details of the major themes and subthemes are described.

#### Real-Time Peer Social and Professional Support

One of the advantages of a mobile phone-based healthy lifestyle program was the ability to create a virtual environment to share experience and information, and to provide support to study participants. The desire for social support from other participants was the most frequent theme reported. In this theme, participants indicated that they wanted real-time support via the mobile phone. Two types of real-time support were identified in the data: (1) peer support from participants who are similarly engaged in a diet or physical activity program, and (2) professional support from health care providers or researchers. In the focus groups, female participants were more likely than male participants to report the importance of a peer support. The participants tended to regard a mobile virtual community as a peer social network or as means to receive real-time professional support. Each subtheme is described below.

##### Peer social support

The study participants reported that a mobile phone program could provide a home base for a real-time peer social support environment. Because individuals can access their mobile phones at their convenience, it is much easier to connect to each other and share their experiences, feelings, and obstacles. They discussed ways in which the phone could act as a virtual companion, allowing them to reach out to others who are also struggling with their diet and exercise challenges, in effect creating a peer social network. The views below exemplify this virtual environment for peer support and social networking:

30-year-old woman Because it comes in as the companion-type thing. It’s like people who understand what you’re going through or you can share and you don’t necessarily have to say,“Hey, I’m ___.”You know what I’m saying?

30-year-old woman ...you also have a forum where you can share with other people who have similar struggles, likes/dislikes, you know, it gives you an outlet so that you can express yourself or what you’re going through, you know?

31-year-old woman ...its easier to do with somebody, or whatever, you know and having a group of people that you are friends with, meeting people, you know, that are in the same situation and having that support network—calling each other up, you know,“How are you doing? Are you still dieting? What did you eat today? I ate this.”

However, participants also seemed to value traditional face-to-face social support combined with a mobile or virtual peer social network. For example, as one woman participant, 58 years old, explained:

59-year-old woman This would work really well for me if about once a month I was in a group like this with other women who got together to talk about their experience of what it was like to have this come over their cell phones. I think if it was just done in a vacuum it wouldn’t—it wouldn’t work as well.

Interestingly, no participant talked about accessing support through existing open social networking platforms, such as Facebook or Twitter. Their desire focused on interacting with people who are in the same healthy lifestyle program.

##### Professional support

Some participants indicated that they also wanted the mobile phone program to act as a connector for professional advice, or as a sponsorship program, such as Alcoholics Anonymous (AA). This subtheme differs from the previous subtheme of peer support and social networking because in this case, the participants tended to be seeking support and encouragement from health care professionals or individuals explicitly defined as “sponsors.” The participants reported that a mobile phone program that included real-time automated messages and feedback could serve as an access point to research staff or health care providers for private virtual consultations about their own progress. The following views exemplify their perspectives:

Having a cell phone and being monitored, kind of have a sponsor. Like a little AA.

37-year-old woman It would be great if—you know they have 1-800-NoButts for smokers who want to quit, I would love to get encouragement over the phone without dealing with a big group of people where you get all embarrassed that you have to lose weight and they know how much weight you’ve lost. You just talk to someone over the phone and it’s totally confidential and they’re like, “Good job, you did great.” This is—try to do this tomorrow. It would be really cool.

Moreover, in order to enhance this professional support or sponsorship component, feedback and 2-way communication are essential components of the program.

42-year-old man I could see getting a text message that said,“Look at your pedometer now. How many steps have you taken?”and you respond and tell them how many steps you’ve taken. And if it comes at the same time every day, then you know somebody’s paying attention, and that would be a motivation, but it isn’t nagging’cause it’s a question. It’s just saying,“How many steps have you taken?”

#### Tailoring of Timing, Frequency, and Content of Messages in Mobile Phone Programs

The mobile phone program should be modified to participants’ own needs. Tailoring of the program was another of the most frequently reported needs among the participants. A mobile phone healthy lifestyle program needed to be flexible enough for them to adapt their own lifestyle and routine, and not dictate their daily activities.

Individual lifestyles vary among people; thus, a mobile phone program should be adaptable to individual needs. In particular, it is important that participants receive both scheduled messages and just-in-time messages timed to meet specific needs. Just-in-time messages can be the most effective message for a participant to change their eating and physical habits. One participant stated that a just-in-time message of “put that candy bar down” would be helpful to her at the time of day when her will power often weakens. Below are examples of participants discussing the importance of tailored, scheduled messages to promote a routine:

37-year-old woman ...you have your timeframe and your availability time. So, you know, if the phone rings and it’s not between 8:00 and 10:00 or something like that on that availability time and then (if it isn’t in the designated time frame) you ignore it.

37-year-old woman Staying on the routine. At a certain time, you know, I can expect to get this text at ten o’clock on Monday every Monday. I know they’re going to text me ten o’clock Monday and you know that is happening.

Frequency of message delivery was also an issue that participants wanted to tailor. Here is one participant’s suggestion for such message tailoring:

37-year-old woman And at one set time a day, or so many times a week the person can—like whenever they sign up, okay, I want my reminders or my texts every day. You know, different people like different stages so they should be able to have that if they need it more or less.

In terms of message content, participants suggested that messages address individual weaknesses. Messages that are tailored to the individual by addressing that individual’s weaknesses are more useful than those that are generalized. As one participant noted:

60-year-old woman Every aspect of our lives reflects our attitudes toward food and without taking that into consideration, just saying, you know,“Eat this”or“Don’t eat that,”that’s just not going to work.

#### Combination of Motivations, Self-Monitoring, and Goal Setting

Participants talked about 4 main components that would motivate them to engage in dietary changes and increasing physical activity. These motives are primarily internal, as they were based in their own personality. The 4 motives identified were competition (with self and/or others in the program), rewards (internal and external), feeling good about themselves, and facing life-threatening illness.

57-year-old man But you know, it’s sort of like competing with yourself. I started taking my blood pressure a couple of years ago and, um, I find myself wanting to get better blood pressure. I think it’s real low-key. It’s real low-key. But when I get a good blood pressure, I go,“That’s alright!”I sort of pat myself on the back, and when I get high blood pressure, I look for excuses.

50-year-old woman Yeah, that would be, like you said, a good idea for us to come back and show our growth and whatever we did and how...—and that’s where the reward comes—see our group who’s the best, who’s done the best, you know what I mean?

49-year-old woman I think, one, if it was like really fun or competitive. I think that would motivate me. And I think the second thing that would motivate me is if I had a like life-threatening illness where I had to exercise or I would decline or whatever. But I think those are the only two instances. It has to be really fun or—or you’re going to die unless you do it.

37-year-old woman I was thinking more along the lines of, you know, monthly (goals). So like you can have your daily goal set...You know, like one day, okay—well, I didn’t get the star three days this week. But if you got the star the rest of the month, you see what I’m saying you would get some type of gift card or, you know, something like that.

The combination of self-monitoring and realistic goal setting also acted as a means of motivation, and participants responded to the notion of having a diary as a way to see their progress or problems. These diaries need to be easy to use or, as one participant stated, not “arduous,” but they liked the idea of being able to complete it in real time and to consult it as needed. This self-monitoring tool could then assist participants in setting goals and making decisions about diet and exercise.

55-year-old woman Just keep it with you and consult it before you pig out for dinner and say, “Well I can’t have that—can’t have that! you’ve already reached my— I can only have 500 calories for this meal.”

These goals also need to reflect the ways in which individuals are motivated to reach for goals.

Realistic goals that are set for individuals are necessary for them to stay motivated and to remain in the program. As one participant noted:

36-year-old man It’ll help that you know, like I say, at the end of the week you can look at your input and you can, you know, give yourself a personal goal. Okay, well, it’s right here, I want it to be up here next week. And you can see where you actually need to go and what you need to do to get there.

#### Potential Barriers and Sustainability of Mobile Phone Programs

Participants discussed potential barriers to both initially engaging with the program and sustaining this engagement over time. Additionally, they provided 3 subthemes for potential barriers and sustainability of the mobile phone program: (1) fear of failing, (2) age and mobile technologies, and (3) loss of interest over time.

##### Fear of failing

One of the most frequently reported potential barriers in the program was that some individuals may not participate in the program or may drop out from the program due to fear of failing to meet goals. This fear of failing was exemplified in discussions of feeling guilty and feeling like a failure; however, participants also addressed ways to mitigate this fear of failing that included positive messaging and setting multiple goals. The following statements highlight this subtheme:

62-year-old woman I really think depending on what you say in that [message] would make a huge difference. I mean if it is very positive and if it’s reinforcement, positive reinforcement, yes. But if you say,“Did you meet your goal this week?”then I’m going to be full of guilt, you know what I mean.

60-year-old woman For me it would be like—I would need more aspects of my life incorporated. Say, for instance, I talk to you this week that next week I want to do such-and-such and such-and-such. And then you call me up and you say,“How about doing such-and-such and while you’re at it—you know, While you’re doing it you’re getting this much exercise or you could do this instead of taking the bus.” Incorporate it in my life so if I fail in one area I haven’t necessarily failed in another area and I am kind of in a way feeling like a failure.I think that’s why I could never do a diet.

To minimize participant’s fear of failing and to motivate participants to change their lifestyle, the program should be positive, and feedback and messages should be encouraging and nonjudgmental. As one participant suggested:

62-year-old woman It has to be a really warm human person that’s not going to sit there and judge you.

##### Age and Mobile Technologies

Some participants reported that they were not comfortable using mobile technologies. Most of these participants were older and had limited experience in using mobile technologies. They indicated that, due to this lack of experience, older participants might view the program as non-user-friendly or too complicated to use. Two women in their 60s stated:

62-year-old woman There might be an issue here too with the age.I mean you people really—they have these machines down, you know. They do it in their sleep, you know, text. But there might be a hurdle for people who are older and there might be some fear around—I mean I still can’t text. I mean I’m lucky when I can text correctly. And I work for software companies.

69-year-old woman I’m not tech savvy, so, I’m from the “old school” and I hate the cell phones my children give me.

In addition, some participants did not own a mobile phone or preferred not to use one because of financial reasons or because of its intrusiveness in their lives. Several participants commented on the cost of the cell phone within their limited budget, one stating: “I’m not paying $40 a month for telephone service.” However, others indicated that they felt that cell phones disrupted their lives, as the 2 quotes below exemplify:

65-year-old woman I don’t own a cell phone. I have an aversion to being on call.

65-year-old woman Could it be the same thing? See, you’re on call, you’ve got to answer to this damn phone. I don’t know, I’m probably in the minority.

##### Loss of Interest Over Time

Another identified potential barrier was that participants who were initially motivated to be involved in the program could lose interest over time. The participants reported that they might delete a message without reading it, or ignore a message if they were no longer excited about the program or no longer valued the messages that they received.

I get something called N___. At first when I got it—it’s a download every day—I’d look at it and be interested.Now I mean I’ve got to delete, I have about five hundred that I haven’t read.

59-year-old woman I know that I get like breaking news. When I first got it I read every one of them and now it’s—I mean now it has to be“President dies”or something before I’m interested.

59-year-old man ...they immediately text message you and I got so many text messages I just stopped reading, I just start deleting.

To avoid this barrier, messages and feedback should not be dull or predictable in pattern. Also, tailored timing, components, and frequency of messages may help to maintain participants’ interest in the program.

## Discussion

Only a few focus group studies have been performed to understand participants’ needs and expectations for a mobile phone program as a process of program development for the prevention of chronic illnesses [8,11,12]. Participants in this study provided valuable data to aid in the development of specific components of a mobile phone program that would motivate individuals with prediabetic conditions to increase their physical activity and improve diet-related behaviors.

The themes we identified were (1) having real-time peer social and professional support, (2) tailoring timing, frequency, and content of messages in mobile phone programs, (3) combining motivations, self-monitoring, and goal setting, and (4) identifying potential barriers and sustainability of mobile phone programs. These findings suggest that creating a mobile virtual community could be effective in engaging and sustaining participants in lifestyle change programs. This virtual community acts as a closed social network that can provide both peer and professional support to participants. It is flexible enough to provide messages and self-monitoring that are tailored to individual participants and address barriers to participation (see Multimedia Appendix 2 for the components of a mobile virtual community).

Participants indicated that the primary component of a successful mobile phone-based healthy lifestyle program was real-time peer social and professional support that would create a mobile virtual community. Through this mobile virtual community, participants could create a peer social network that would support their efforts to change their diet and physical activity and receive immediate feedback from health care providers, a designated sponsor, or a peer [13,14]. Participants indicated that they would be comfortable sharing their experiences and struggles with others in their virtual community, but they would not be comfortable doing so with people outside the created virtual community. Contrary to Greene et al., participants in our study indicated that they wanted a closed social network rather than an open one [15]. Although Greene et al. reported that Facebook enabled participants to share experiences and to receive answers to their questions or to receive direct feedback from individuals with diabetes, none of our participants mentioned an interest in open social networks such as Facebook [15]. In addition, social support strongly relates to motivations for lifestyle change. Peer social support is well known to be a crucial motivational tool to promote physical activity [16], particularly in women. Since a mobile phone program is very portable and participants can bring it with them wherever they go, they can receive continuous support and be connected to their peers at all times. Thus, participants in this mobile phone program may perceive the virtual mobile community as a beneficial component of the program.

Tailoring of the program is another key component of a successful mobile phone program [17-21]. Frequency, timing, and content of messages, as well as goal reminders, should be tailored to an individual’s needs. Just-in-time messages or feedback at the moment of decision or action play an important role in keeping participants motivated [22]. To monitor participants’ behaviors and send just-in-time messages, the program should have the ability of not only sending messages, but also of receiving reports from participants about their current activities and of providing feedback. A program of this capacity can send real-time, context-aware feedback and messages, and can get feedback in real time. The real-time feedback component of the program that elicits messages before patients take counterproductive actions is important to motivate then to make healthy choices [23]. The program, therefore, must incorporate 2-way messaging. Previous programs using mobile phone technology have focused on using 1-way SMS (text messages), but this may not be sufficient for all participants. Importantly, feedback and goal setting need to be tailored to an individual based on their strength, ability, and barriers, as well as achievement of the previous goal [23,24].

This study reinforces the need for self-monitoring; however, participants also connected this idea with broader motivations for enacting behavioral changes. Participants identified the value of competition to keep one engaged and the importance of some form of reward mechanism. The challenge for a mobile phone program is to incorporate different ways to motivate participants. The program would have to be able to engender a sense of competition, at least within participants that are so motivated, but it would also need to determine effective rewards that can be potentially tailored, both individually and over time.

Participants also identified potential barriers to initiating and sustaining engagement with the program. Fear of failing was one of the major potential barriers addressed by participants. A previous study found that women who used a mobile phone physical activity program perceived the program as a virtual coach to increase their motivation for a healthier lifestyle. Coaching is an effective approach to behavior change, but pressure from a coach or another’s expectation can create fear of failure in the participant [25,26]. To minimize participants’ fears, nonjudgmental approaches, positive reinforcement, and establishing more than 1 short-term goal should be factored into the mobile phone program to mitigate fears of failing.

Age and the potential intrusiveness of mobile phones in people’s lives could also be a barrier to engagement with the program. Older participants expressed concerns about their ability to use the mobile phone technologies, especially texting, and that their lack of technical ability might limit their willingness to participate in the program. Over 25% of the participants had not used a mobile phone in the last month. For some, this was due more to lack of funds than to a dislike of mobile phones. However, a few expressed a rather strong antagonism toward the impact of mobile phones on their way of life. Given this expressed antipathy to mobile phones and the number of nonusers in the focus groups, conducting further research with only nonusers could be warranted to more fully understand this barrier.

In terms of sustaining participation in this type of program, there is limited research that has explored issues around long-term engagement in these types of programs. Participants in this study indicated that they needed to continue to value the program. Suggestions for how to maintain interest over time included keeping the program simple and user friendly. This component may be particularly important to accommodate older adults, people with less education, and people who are unfamiliar with mobile phones.

Additionally, the messages need to be short and varied over time. In addition, a system of rewards needs to immediately reflect their positive behavior change but may also need to change over time. A randomized variety of messages may help to avoid the boredom, and a program tailored to the individual’s needs could help to keep participants motivated. However, more research is needed to understand how these rewards can be incorporated into mobile phone technologies and how to modify them over time. Finally, resistance to using mobile technologies among older individuals is also a potential barrier [27] that needs to be more fully explored. The participants’ suggestions and ideas based on their own experiences are great sources to develop the most effective components of a mobile phone-based healthy lifestyle program.

### Limitations of the Study

The study results should be interpreted within the following limitations. All participants in this focus group were recruited from only 1 geographic location, San Francisco, approximately 74% of the participants were unemployed, and 49% had incomes less than $20,000. Thus, the findings in the study may not be representative of more affluent or more fully-employed populations in the United States.

### Conclusions

Participants from a wide range of age and racial groups expressed interest in a mobile phone-based lifestyle program. A mobile phone-based lifestyle program that incorporates these identified themes may be able to help motivate participants to increase their physical activity and to improve their diet. This study can help researchers to further understand individual needs and expectations of a mobile phone program to motivate and promote healthy lifestyle modifications in sedentary and overweight or obese adults.

## Multimedia Appendix 1

Questions for Focus Groups

## Multimedia Appendix 2

Summary of Focus Group Themes

## Abbreviations

AA: Alcoholics Anonymous
BMI: body mass index
SMS: short message service

## References

[1] American Diabetes Association (2011). American Diabetes Association.

[2] Knowler WC, Barrett-Connor E, Fowler SE, Hamman RF, Lachin JM, Walker EA, Nathan DM, Diabetes Prevention Program Research Group (2002). Reduction in the incidence of type 2 diabetes with lifestyle intervention or metformin. N Engl J Med.

[3] Tuomilehto J, Lindström J, Eriksson JG, Valle TT, Hämäläinen H, Ilanne-Parikka P, Keinänen-Kiukaanniemi S, Laakso M, Louheranta A, Rastas M, Salminen V, Uusitupa M, Finnish Diabetes Prevention Study Group (2001). Prevention of type 2 diabetes mellitus by changes in lifestyle among subjects with impaired glucose tolerance. N Engl J Med.

[4] Sandelowski M (2000). Whatever happened to qualitative description?. Res Nurs Health.

[5] Centers for Disease Control and Prevention (2009). CDC.

[6] Anhøj J, Møldrup C (2004). Feasibility of collecting diary data from asthma patients through mobile phones and SMS (short message service): response rate analysis and focus group evaluation from a pilot study. J Med Internet Res.

[7] Arsand E, Tatara N, Østengen G, Hartvigsen G (2010). Mobile phone-based self-management tools for type 2 diabetes: the few touch application. J Diabetes Sci Technol.

[8] Pinnock H, Slack R, Sheikh A (2007). Misconnecting for health: (lack of) advice for professionals on the safe use of mobile phone technology. Qual Saf Health Care.

[9] Holtzblatt K (2005). Customer-centered design for mobile applications. Personal and Ubiquitous Computing.

[10] Fukuoka Y, Vittinghoff E, Jong SS, Haskell W (2010). Innovation to motivation: pilot study of a mobile phone intervention to increase physical activity among sedentary women. Prev Med.

[11] Boushey CJ, Kerr DA, Wright J, Lutes KD, Ebert DS, Delp EJ (2009). Use of technology in children's dietary assessment. Eur J Clin Nutr.

[12] Halifax NV, Cafazzo JA, Irvine MJ, Hamill M, Rizo CA, McIssac WJ, Rossos PG, Logan AG (2007). Telemanagement of hypertension: a qualitative assessment of patient and physician preferences. Can J Cardiol.

[13] Eagle N, Pentland AS, Lazer D (2009). Inferring friendship network structure by using mobile phone data. Proc Natl Acad Sci U S A.

[14] Jen WY (2010). The adoption of mobile weight management services in a virtual community: the perspective of college students. Telemed J E Health.

[15] Greene JA, Choudhry NK, Kilabuk E, Shrank WH (2011). Online social networking by patients with diabetes: a qualitative evaluation of communication with Facebook. J Gen Intern Med.

[16] Salvy SJ, Roemmich JN, Bowker JC, Romero ND, Stadler PJ, Epstein LH (2009). Effect of peers and friends on youth physical activity and motivation to be physically active. J Pediatr Psychol.

[17] Bull FC, Holt CL, Kreuter MW, Clark EM, Scharff D (2001). Understanding the effects of printed health education materials: which features lead to which outcomes?. J Health Commun.

[18] Dijkstra A, De Vries H (1999). The development of computer-generated tailored interventions. Patient Educ Couns.

[19] Ryan P, Lauver DR (2002). The efficacy of tailored interventions. J Nurs Scholarsh.

[20] Suggs LS (2006). A 10-year retrospective of research in new technologies for health communication. J Health Commun.

[21] Trevena LJ, Davey HM, Barratt A, Butow P, Caldwell P (2006). A systematic review on communicating with patients about evidence. J Eval Clin Pract.

[22] Intille SS (2004). A new research challenge: persuasive technology to motivate healthy aging. IEEE Trans Inf Technol Biomed.

[23] Hurling R, Catt M, Boni MD, Fairley BW, Hurst T, Murray P, Richardson A, Sodhi JS (2007). Using internet and mobile phone technology to deliver an automated physical activity program: randomized controlled trial. J Med Internet Res.

[24] Tufano JT, Karras BT (2005). Mobile eHealth interventions for obesity: a timely opportunity to leverage convergence trends. J Med Internet Res.

[25] Elder JP, Ayala GX, Harris S (1999). Theories and intervention approaches to health-behavior change in primary care. Am J Prev Med.

[26] Sagar SS, Stoeber J (2009). Perfectionism, fear of failure, and affective responses to success and failure: the central role of fear of experiencing shame and embarrassment. J Sport Exerc Psychol.

[27] Charness N, Boot WR (2009). Aging and information technology use: potential and barriers. Curr Dir Psychol Sci.

