# Supplementary material for: Real-Time Social Support Through a Mobile Virtual Community to Improve Healthy Behavior in Overweight and Sedentary Adults: A Focus Group Analysis
Source: J Med Internet Res. 2011 Jul 14;13(3):e49. doi: 10.2196/jmir.1770 (PMC3222182; doi:10.2196/jmir.1770)
Supplement: Supplementary file 1 [file jmir_v13i3e49_app1.pdf]

**Appendix A. Questions for Focus Groups**	
	**Questions for Focus Groups**
**Opening Question**	What is diabetes? What does it mean to you? (probe: do you know anyone with diabetes?)
**Semi-structured format**	**Sample questions / prompts**
Diabetes	How can you prevent diabetes? (probe: knowledge, skills, lifestyle changes). How can you control diabetes?
Weight control	Weight loss: Tell us about your previous experiences in losing weight? (diet, exercise, commercial program and you tried). What stopped you from continuing?
Physical activity	How active are you? (probe: kinds of physical activities you do on a daily basis). Tell us what would motivate you to be more physically active? What keeps you from being physically active? What would it take to keep you physically active?
Diet	What is a healthy diet? Describe your normal eating patterns (when do you eat, what do you eat, how much, environment). What keeps you from always eating a healthy diet? What would motivate you to eat more healthy foods?
Mobile technologies and mobile phone program specific questions*	If you were going to use a mobile phone to motivate people to be physically active or eat a healthy diet, how would you use the phone? (probe: messages--what types of messages would you like? How often and when would you like them? If you were going to get motivational messages via the mobile phone—how would you like to receive them? What kind of contact would you like with researchers, with each other?- show examples of possible messages—ask for their feedback on these messages)

***** The analysis presented in this paper focuses on these program specific questions.
