# Supplementary material for: Real-Time Social Support Through a Mobile Virtual Community to Improve Healthy Behavior in Overweight and Sedentary Adults: A Focus Group Analysis
Source: J Med Internet Res. 2011 Jul 14;13(3):e49. doi: 10.2196/jmir.1770 (PMC3222182; doi:10.2196/jmir.1770)
Supplement: Supplementary file 2 [file jmir_v13i3e49_app2.pdf]

Appendix B. Summary of Focus Group Themes	
A mobile virtual community for a healthy lifestyle program is an environment created to share goals and experiences, exchange information, and provide just-in-time feedback and real time social support via a mobile phone.	
1.	**Social support:** Real time peer or professional social support is a key component of a mobile virtual community in a closed social network. Social support via a mobile phone may also play a role as a virtual companion or connector.
2.	**Tailoring a program:** A cycle of tailoring the timing, frequency, content of messages and providing real time feedback provides coaching. Assessing each participant's needs and preferences is important.
**3.**	**Motivation:** A mobile virtual community can provide different motivators, such as competition with self and/or peers and provides internal and/or external rewards. Realistic goal setting with self-monitoring tools can also motivate participants to remain in this program.
**4.**	**Potential barriers and sustainability of such programs:** Fear of failing to meet the goals or expectations of others could be a barrier. Given the rapid growth of mobile technologies, applying new technologies can be another barrier, in particular with older participants. Initial motivation to be involved in the program can diminish over time.
